# Diet or additional supplement to increase potassium intake: protocol for an adaptive clinical trial

**DOI:** 10.1186/s13063-022-06071-9

**Published:** 2022-02-14

**Authors:** Swapnil Hiremath, Dean Fergusson, Greg Knoll, Tim Ramsay, Jennifer Kong, Marcel Ruzicka

**Affiliations:** 1 Division of Nephrology, The Ottawa Hospital and Department of Medicine, University of Ottawa, Ottawa, Ontario Canada; 2 Clinical Epidemiology Program, The Ottawa Hospital Research Institute, Ottawa, Ontario Canada; 3 Methods Centre, The Ottawa Hospital Research Institute, Ottawa, Ontario Canada; 4 Division of Nephrology, Ottawa Hospital Research Institute, Ottawa, Ontario Canada

**Keywords:** Hypertension, Potassium, Supplementation, Blood pressure, Diet, Lifestyle, Counselling

## Abstract

**Background:**

High blood pressure is the leading cause of cardiovascular disease worldwide. The prevalence of high blood pressure is steadily rising as the population grows amongst older adults with the ageing population. Therapeutical treatments are widely available to decrease blood pressures, in addition to many lifestyle options, such as dietary changes and exercise. There is a marked preference amongst patients, as reiterated by Hypertension Canada, for more research into non-therapeutic methods for controlling blood pressure or to reduce the burden of taking many pills to control high blood pressure. Indeed, effective options do exist, especially with diet, specifically decreasing sodium and increasing potassium intake. Current public health outreach primarily focusses on sodium intake, even though potassium intake remains low in the Western world. Excellent data exist in published research that increasing potassium intake, either via dietary modification or supplements, reduces blood pressure and reduces risk of cardiovascular outcomes such as stroke. However, the advice most often provided by medical professionals is to ‘eat more fruits and vegetables’ which has little impact on patient outcomes.

**Methods:**

We propose to do a clinical trial in two stages with an adaptive trial design. In the first stage, participants with high blood pressure and proven low potassium intake (measured on the basis of a 24-h urine collection) will get individually tailored dietary advice, reinforced by weekly supportive phone/email support. At 4 weeks, if there has not been a measured increase in potassium intake, participants will be prescribed an additional potassium supplement. Testing will be conducted again at 8 weeks, to confirm the efficacy of the potassium supplement. Final measurements will be planned at 52 weeks to observe and measure the persistence of the effect of diet or additional supplement. Concurrent measurements of sodium intake, blood pressure, participant satisfaction, and safety measures will also be done.

**Discussion:**

The results of the study will help determine the most effective method of increasing potassium intake, thus reducing blood pressure and need for blood pressure-lowering medicines, and at the same time potentially increasing participant satisfaction. The current guidelines recommend changes in diet, not a potassium supplement, to increase potassium intake; hence, the two-stage design will only add supplements if the most rigorous dietary advice does not work.

**Trial registration:**

This study has been registered on ClinicalTrials.govNCT03809884. Registered on January 18, 2019

**Supplementary Information:**

The online version contains supplementary material available at 10.1186/s13063-022-06071-9.

## Background

Hypertension is the leading cause of death and disability in Canada and globally. Apart from a wide choice of pharmacological agents, multiple lifestyle modifications, in particular an increase in potassium intake by diet or as a supplement, have been shown to be efficacious in reducing blood pressure (BP) [1–3]. An increase in dietary potassium is recommended by the World Health Organization [4], the American Heart Association/American College of Cardiology [5], and Hypertension Canada [6, 7]. Despite this, potassium intake remains stubbornly low in western world, including in Canada. Similar to the relative slowness in behaviour change with reducing sodium, the reason why trials of efficacy may not have translated into change is that the interventions in the trials of dietary modification have not been feasible (including supervised intake of meals and/or provision of meals) to be used in clinical practice. In contrast to behavioural modification required for increasing potassium intake in food, namely change in dietary habits, addition of potassium supplements might be an easy to implement alternative [8, 9]. The advocacy of potassium supplements has not made it into any guidelines despite robust data of their efficacy and the public enthusiasm for consuming health supplements.

Increasing potassium intake itself is a robust, but often overlooked, means of decreasing BP [9, 10]. The effect of increasing potassium on BP is seen at low as well as high sodium intake, for example in the Dietary Approach to Stop Hypertension Sodium (DASH Sodium) trial, the effect of the potassium rich DASH diet was − 5.0/− 2.5 mm Hg at high sodium intake and − 2.2/− 1.0 at low sodium intake [11] . Increasing dietary potassium intake has an inverse association not just with BP but can impact cardiovascular disease. Systematic reviews and meta-analyses of randomized controlled trials on the effect of increased potassium intake on BP show significant decreases in systolic and diastolic BP with increased potassium intake [8–13]. Globally, hypertension is the leading risk factor for mortality, accounting for 13% of death, and also being the leading cause of disability worldwide, according to the Global Burden of Disease studies [14–16]. Research on lifestyle habits to reduce blood pressure, without involving pharmacotherapy has been identified as a top research priority by Hypertension Canada [1].

Advocating high potassium intake either in the form of diet or as use of a potassium supplement to the diet may pose a risk in certain populations. However, except in individuals with advanced chronic kidney disease or other conditions impairing renal potassium handling, adaptive mechanisms are triggered that allow for excretion of excess potassium in the urine [17–19]. Thus, increasing potassium intake, as part of the diet or as a supplement, has little adverse effects in most populations [9, 13]. In this adaptive trial, we will test the effectiveness of dietary counselling, followed by additional potassium supplementation in those in whom dietary counselling is ineffective. The objective of this study is to determine an effective strategy for increasing potassium intake in hypertensive individuals with low potassium intake.

## Methods

### Study design and setting

**Fig. 1:**
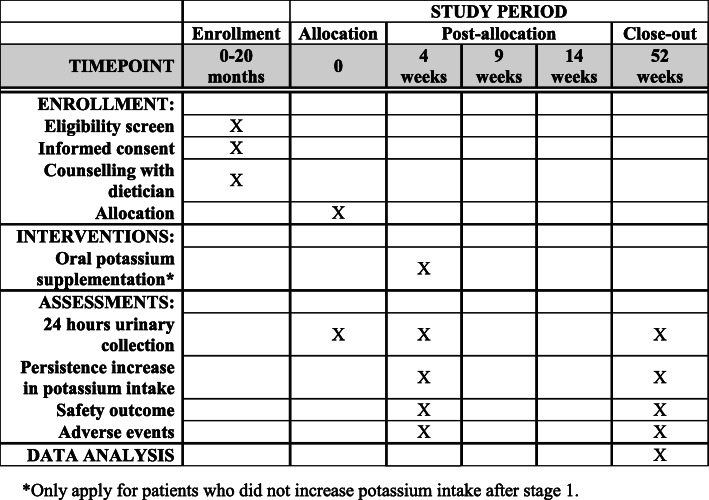
Stage 1 study period content for all participants (stage 1), including the schedule of enrollment, interventions, and assessments for the Diet or Additional Supplement to Increase Potassium study

**Fig. 2:**
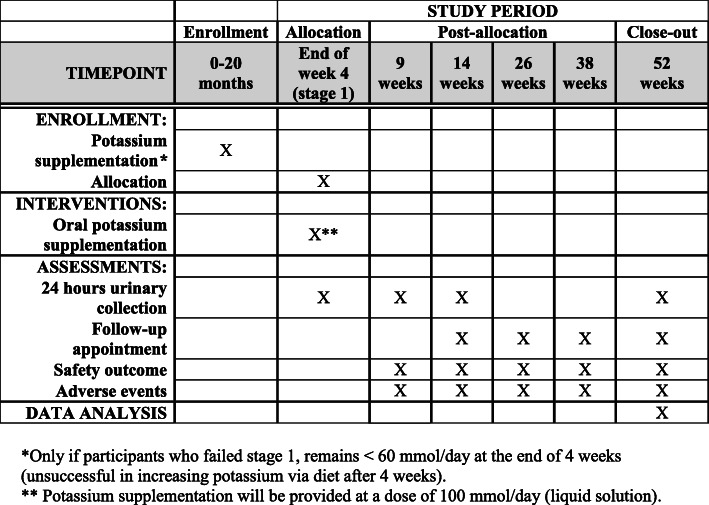
Stage 2 study period content for participants who failed stage 1, including the schedule of enrollment, interventions, and assessments for the Diet or Additional Supplement to Increase Potassium study

The study design is a single-centre, single arm clinical trial, with an adaptive design of two possible sequential interventions. For the dietary advice, this is outside the phases which apply to pharmacological trial regulatory description. The potassium supplement aspect could be considered as a phase 2, since, though approved for other indications, they have not been approved for hypertension. Focused and individualized dietary counselling to increase potassium intake will be the initial intervention amongst hypertensive individuals with low potassium intake. The aim of the study is to find the most effective intervention to increase potassium intake in this population. Since diet is the only recommendation in current clinical practice guidelines, this will be the initial intervention. Only in participants who do not achieve an increase in potassium intake, as measured on the basis of her 24-h urine potassium, as described below, will a potassium supplement be added. From a safety perspective, this was thought to be the most suitable study design. A comparison of a potassium supplement with a diet might result in a change in diet in patients randomized to a supplement, with possible safety issues in terms of hyperkalemia. Hence, the study design is a two-stage intervention, of dietary counselling, with the second stage of supplement only added to those in home the dietary counselling has been insufficient in increasing potassium intake (Figs. 1 and 2).

This study and patient recruitment will take place in a referral hypertension clinic at a tertiary care teaching hospital. Patients with difficult to control hypertension are referred to this clinic, and all patients undergo evaluation of their sodium and potassium intake with a 24-h urinary collection. The study coordinator will obtain written informed consent and to ensure that all necessary information is obtained. During the consent process, the study coordinator will ensure that there are no overtures of coercion, duress, or undue incentive. On the consent form, participants will also be asked if they agree to use their data should they choose to withdraw from the study.

### Study population and timeline

In this trial, all patients with low dietary potassium intake will be eligible for enrolment. The study coordinator will screen patient for eligibility and obtain informed consent. Patients known to be at risk for hypokalaemia or hyperkalaemia will be excluded. The estimated duration for this study is 4 years.

Inclusion criteria:

(1) Hypertension (either on treatment, any level of BP; or not on treatment with an ambulatory blood pressure monitoring (ABPM) with daytime systolic blood pressure (SBP) > 140 or diastolic blood pressure (DBP) > 90)

(2) Aged 18 and greater

(3) 24-h urine potassium < 60 mmol

Exclusion criteria:

(1) Serum potassium < 3.3 or > 5.1 mmol/L

(2) Glomerular filtration rate (GFR) < 45 ml/min/1.73 m^2^

(3) Primary hyperaldosteronism

(4) Pregnancy

(5) Psychiatric disorder which, in the opinion of the investigator, would interfere with the study, or inability to give consent

(6) Severe liver disease

(7) Metabolic alkalosis (HCO_3_ > 32 mmol/L)

(8) Exclude patients who need to be started on renin-angiotensin-aldosterone blockade in the next 3 months

(9) Gastrointestinal disorder (delayed gastric emptying, dysphagia, gastric/duodenal/oesophageal ulcers)

### Interventions

#### Stage 1: Counselling

All enrolled patients will undergo a 1:1 counselling with a registered dietitian (with possible inclusion of family members, as appropriate). The dietitian will undertake an assessment of the comorbidities (e.g. diabetes), dietary intake, and dietary habits (e.g. eating out, food preparation, socio-cultural aspects) and provide an individually tailored strategy to increase potassium in the diet. Secondly, on a weekly basis, the dietitian will contact the patient by telephone or electronically (as preferred by the patient) to reinforce the advice and provide support and advice as necessary. Patients who are successful in increasing potassium to desirable levels at 4 weeks (see the “Outcomes” section) will continue to have follow-up for one more year.

The counselling will be individualized and focused on addition of food ingredients or items to increase potassium intake, rather than a complete overhaul of the participant lifestyle.

#### Stage 2: Potassium supplementation

Patients who are not able to successfully increase their potassium intake at 4 weeks with dietary counselling will be enrolled into stage 2. They will receive oral potassium supplementation in the form of 50 to 100 mmol of potassium citrate (25 to 50 ml of the liquid elixir based on the achieved 24-h urinary potassium). For those whose urinary 24-h potassium remains < 60 mmol/day, the dose of the supplement will be 100 mmol/day, and for those whose 24-h urinary potassium is between 60 and 90 mmol/day, the dose will be 50 mmol/day. In the DASH trial, the diet itself provides 120 mmol/day of potassium; however, the average measured 24-h urinary potassium was 2757 mg/day (~ 71 mmol/day). Hence, we are aiming for a higher dose of supplement to achieve the target outcome.

This dose of potassium is well tolerated in hypertension (reported adverse events, at < 1% frequency and not different from placebo, are change in bowel habits, belching and flatulence, and abdominal cramps with the current wax matrix-based and microencapsulated or coated microcrystal-containing preparations with extended release characteristics) [9, 20]. There are no reports of hyperkalemia reported in trials using this dose in a similar population [9, 13]. Potassium at this dose and formulation has excellent (> 90%) bioavailability [20]. This dose of potassium also should be effective in increasing urinary potassium to desired levels. There is also no comparator in this study since it is not a randomized controlled trial. Since potassium is well tolerated in hypertension, there will be no special criteria for discontinuing or modifying allocated interventions.

### Outcomes

The primary outcome will be a successful increase in potassium intake to > 90 mmol/day as estimated from the 24-h urinary sample at 4 weeks. The secondary outcomes are persistence of increase in potassium intake (to > 90 mmol/day) at 1 year. For examination of safety, the following outcomes will be specifically examined: hyperkalemia (as defined as a serum potassium > 5.1 mmol/L) at 4 weeks after initiation of dietary counselling, at 1 and 4 weeks after starting potassium supplements, 1 week after dose escalation/dispensing, and at 12 months in everyone and gastrointestinal side effects (change in bowel habits, belching and/or flatulence; abdominal pain or cramps). Additional patient-reported adverse effects will also be measured and reported. Serious adverse events will be captured and reported as per regulatory requirements.

### Blinding

The trial design is open label, given that behavioural change is difficult to blind. However, numerous safeguards will be in place to minimize actual bias in the data collected.

#### Blinded assessment of outcomes

The primary outcome in this trial is a change in potassium intake, as measured by 24-h urinary potassium. This is an objective measure, however, and the laboratory personnel measuring the values will not be aware of which intervention is ongoing when they perform the measurement.

#### Ascertainment and follow-up plan

In order to minimize ascertainment bias, all patients, irrespective of the group, will have similar measurement schedule. Nevertheless, we will study the frequency of loss to follow-up and reasons thereof.

### Measurements

The dietary intake of potassium will be assessed using 24-h collection of urine. There is a very close relationship overall between urinary potassium excretion and dietary potassium intake. There is a circadian variation [21] (more potassium excretion in the day than at night) which will be overcome with a 24-h urine collection. Twenty four-hour urinary sodium and creatinine (to assess for accuracy of collection) will also be measured for all participants at baseline, 4 weeks, and 52 weeks (end of study). In addition, participants who enrol into stage 2 will have an additional 24-h urinary collection and set of measurements at 4 weeks after initiation of the potassium supplement/dose escalation. Blood will also be collected at the same time points for measurement of kidney function and electrolytes (creatinine, sodium, potassium, total CO_2_, chloride). As an additional safety measure, serum potassium will be measured 1 week after starting potassium supplementation, 1 week after dose escalation, and 1 week post each re-dispensing visit. Blood pressure will be measured using automated oscillometric measurements and 24-h ambulatory monitoring. Additional assessment of adherence to supplement (apart from 24-h urine measures) will be made on the basis of returned pills/bottles. Patients satisfaction will be assessed using a simple 3 question survey (see supplementary material). Furthermore, all collected samples will be sent over to the laboratory for analyses. Any leftover samples will be discarded as medical wastes. There are no plans for subsequent or ancillary analyses for the future studies.

### Sample size and analytical plan

The study goal is to estimate the success rate of the dietary intervention, as well as the success rate of the two-stage approach. Dietary counselling alone will not exceed this degree of increase which was seen with supervised intake and meal provision, but there are no robust data to support an estimate of its effect. However, potassium supplementation at the dose proposed would very likely result in achieved potassium intake of > 90 mmol/day in all participants. A sample size of 100 participants will allow us to able to estimate both success rates to within a margin of error of at most 5%. At 4 weeks, there should be little loss to follow-up; however, we estimate this to be about 20% to be conservative. Thus, this trial will enrol 120 participants. Furthermore, there are no plans for additional analyses including subgroup. Multiple imputation will be done for any missing data.

The primary outcome is a simple proportion of the participants who are able to achieve an increase in potassium intake. Secondary outcomes of the proportion of participants with persistent adequate potassium intake at 52 weeks, and safety outcomes, will also be reported as proportions. For the secondary outcomes of change in sodium, potassium, and blood pressure, the mean differences will be calculated and reported, and a paired *t*-test will be used to compare for statistical significance. There will be no interim analysis.

The proportions will be summarized as absolute numbers and percentages. Continuous outcomes will be reported as mean (and 95% confidence intervals). A *p* value of < 0.05 will be used for the paired comparisons of the continuous outcomes (i.e. secondary outcomes of change in sodium, potassium and blood pressure).

### Adverse events

The qualified investigator will follow adverse events with start dates occurring any time after informed consent is obtained until 7 days (for non-serious adverse events) or 30 days (for severe adverse events) after the last day of study participation. At each study visit, the investigator (or designee) will inquire about the occurrence of adverse events (AE)/serious adverse events (SAEs) since the last visit and record in participant research record and case report form. Events will be followed for outcome information until resolution or stabilization. This trial is a low risk, non-invasive intervention, with minimal adverse events anticipated and hence anticipation of low rate of loss to follow-up. Nevertheless, hypertension is a chronic disease, and it is possible that the complete 1 year follow-up will not occur for all patients and has a built in 20% loss in the sample size estimation.

### Data safety monitoring board

This study will be monitored by an independent data safety monitoring board (DSMB), consisting of a clinical epidemiologist, a nephrologist, and a biostatistician. The DSMB will be immediately informed of any SAEs which may potentially be related to the study intervention. Other SAEs will be reviewed during regular DSMB meetings. Interim reports, prepared by the data management team for the study, for review by the DSMB will include data on recruitment, compliance, adverse effects, baseline comparability, and treatment comparisons. An agreed upon review package which contains the appropriate data summary by treatment will be provided by the study statistician for the purposes of these reviews.

### Study management

A trial management team involving principal investigators (SH and MR), three co-investigators (DF, GK, TR), and one of study coordinator and research assistant will review, implement, and supervise all aspect of this study. The responsibility for study design, data collection, interpretation, and writing of the manuscript and decision to submit the report for publication reside with the research team and not the funding sponsor. The Project Management Group will meet every 6 months to review trial conduct.

Access to medical records and study data will be limited to authorized personnel listed on the study delegation log or permitted by the study agreement. All data collected during the course of research will be kept strictly confidential and accessed by members of the study team. Access to electronic data will be password protected and auditable, electronic data will be stored on a hospital network with firewall and security back-up measure in place, and paper copies of the study data will be stored securely in locked cabinets and in locked offices.

Participants will be allocated an individual trial identification number and will be store in a secure database. Only the research team will have access and control over the trial dataset. The final trial data for this protocol can be available upon request. The datasets analysed during the current study and statistical code are available from the corresponding author on reasonable requests, as is the full protocol. In addition, there is no anticipated harm and compensation for trial participation. All participants will continue to receive standard of care post trial.

## Discussion

There is unequivocal evidence on the efficacy of potassium intake in lowering blood pressure and subsequent cardiovascular outcomes. However, the trials have not resulted in change in practice due to a lack of data on the most effective methods to implement this change. Our adaptive trial design, with sequential intervention of the dietary counselling, followed by additional supplementation in those in whom diet alone is ineffective, represents an easy to implement intervention. This might bridge the efficacy to effectiveness implementation gap. A 5-mm Hg decrease in systolic blood pressure, as reported with additional potassium effect in meta-analyses, translates into one less antihypertensive medication. Thus, the results of this trial have the potential for a very large scale impact at a public health, as well at an individual level. Lifestyle habits to improve blood pressure and decrease medication burden have been already identified as high level research priorities by patients in work done by Hypertension Canada and others. We hope the results of this trial, once completed and reported, will provide useful and actionable information for public health in this sphere.

## Trial status

The trial has received ethics approval from the Ottawa Health Science Research Ethics Board (OHSN-REB) on February 5, 2019, reference number 20180873-01H. Trial screening and recruitment has begun in January 2020, with anticipated completion in 2023. Protocol version and date: January 21, 2022. The datasets analysed during the current study and statistical code are available from the corresponding author on reasonable request, as is the full protocol.

## Supplementary Information

**Additional file 1:.** Supplementary material. Three question survey at 52 weeks

## Data Availability

Supplementary material including a simple 3 question survey.

## Abbreviations

BP: Blood pressure
ABPM: Ambulatory blood pressure monitoring
DSMB: Data safety monitoring board
GFR: Glomerular filtration rate
SBP: Systolic blood pressure
DBP: Diastolic blood pressure
TOH: The Ottawa Hospital
AE: Adverse events
SAE: Severe adverse events
