# Supplementary figures and images for: Diet or additional supplement to increase potassium intake: protocol for an adaptive clinical trial
Source: Trials. 2022 Feb 14;23:147. doi: 10.1186/s13063-022-06071-9 (PMC8845348; doi:10.1186/s13063-022-06071-9)

Supplementary material. Three question survey at 52 weeks.


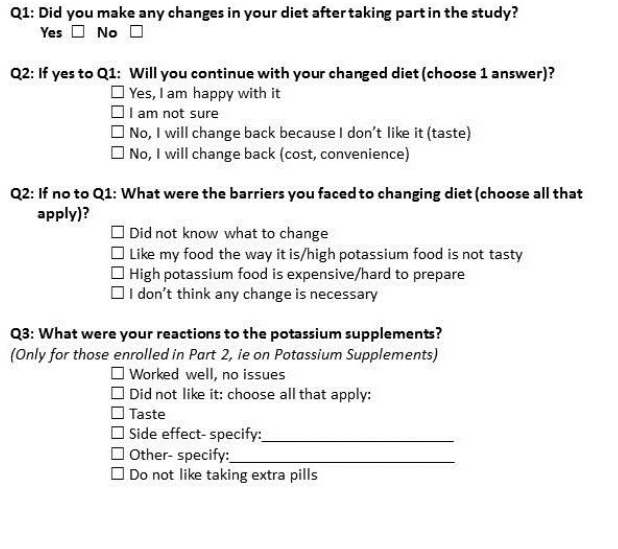

Supplement: Supplementary file 1 — Additional file 1:. Supplementary material. Three question survey at 52 weeks [file 13063_2022_6071_MOESM1_ESM.docx]
